# Laboratory evolution of *E. coli* with a natural vitamin B_12_ analog reveals roles for cobamide uptake and adenosylation in methionine synthase-dependent growth

**DOI:** 10.1128/jb.00284-24

**Published:** 2025-01-28

**Authors:** Kenny C. Mok, Zachary F. Hallberg, Rebecca R. Procknow, Michiko E. Taga

**Affiliations:** 1 Department of Plant & Microbial Biology, University of California Berkeley 118549 , Berkeley, California, USA; University of Virginia School of Medicine, Charlottesville, Virginia, USA

**Keywords:** pseudocobalamin, vitamin B_12_, cobalamin, cobamide, corrinoid adenosyltransferase, corrinoid uptake, methionine synthase, bacterial evolution

## Abstract

Bacteria encounter chemically similar nutrients in their environment, which impact their growth in distinct ways. Among such nutrients are cobamides, the structurally diverse family of cofactors related to vitamin B_12_ (cobalamin), which function as cofactors for diverse metabolic processes. Given that different environments contain varying abundances of different cobamides, bacteria are likely to encounter cobamides that enable them to grow robustly and also those that do not function efficiently for their metabolism. To gain insights into how bacteria might respond under the latter conditions, we performed a laboratory evolution of a cobamide-dependent strain of *Escherichia coli* with pseudocobalamin (pCbl), a cobamide that *E. coli* uses less effectively than cobalamin for MetH-dependent methionine synthesis. Propagation and sequencing of nine independent lines identified potential genetic adaptations in cobamide-related genes that improved growth with less-preferred cobamides. We constructed targeted mutants to validate these findings and found that increasing the expression of the outer membrane cobamide transporter BtuB is beneficial during growth under cobamide-limiting conditions. Unexpectedly, we also found that overexpression of the cobamide adenosyltransferase BtuR confers a specific growth advantage with pCbl. Characterization of the latter phenotype revealed that BtuR and adenosylated cobamides contribute to optimal MetH-dependent growth. Together, these findings improve our understanding of how bacteria expand their cobamide-dependent metabolic potential.

**IMPORTANCE:**

In nature, bacteria commonly experience fluctuations in the availability of required nutrients. Thus, their environment often contains nutrients that are insufficient in quantity or that function poorly in their metabolism. Cobamides, the vitamin B_12_ family of cofactors, are ideal for investigating the influence of nutrient quality on bacterial growth. We performed a laboratory evolution experiment in *E. coli* with a less-preferred cobamide to examine whether and how bacteria can improve their growth with less ideal nutrients. We found that overexpression of genes for cobamide uptake and modification are genetic adaptations that improve growth under these conditions. Given that cobamides are key shared metabolites in microbial communities, our results reveal insights into bacterial interactions and competition for nutrients.

## INTRODUCTION

**Fig 1:**
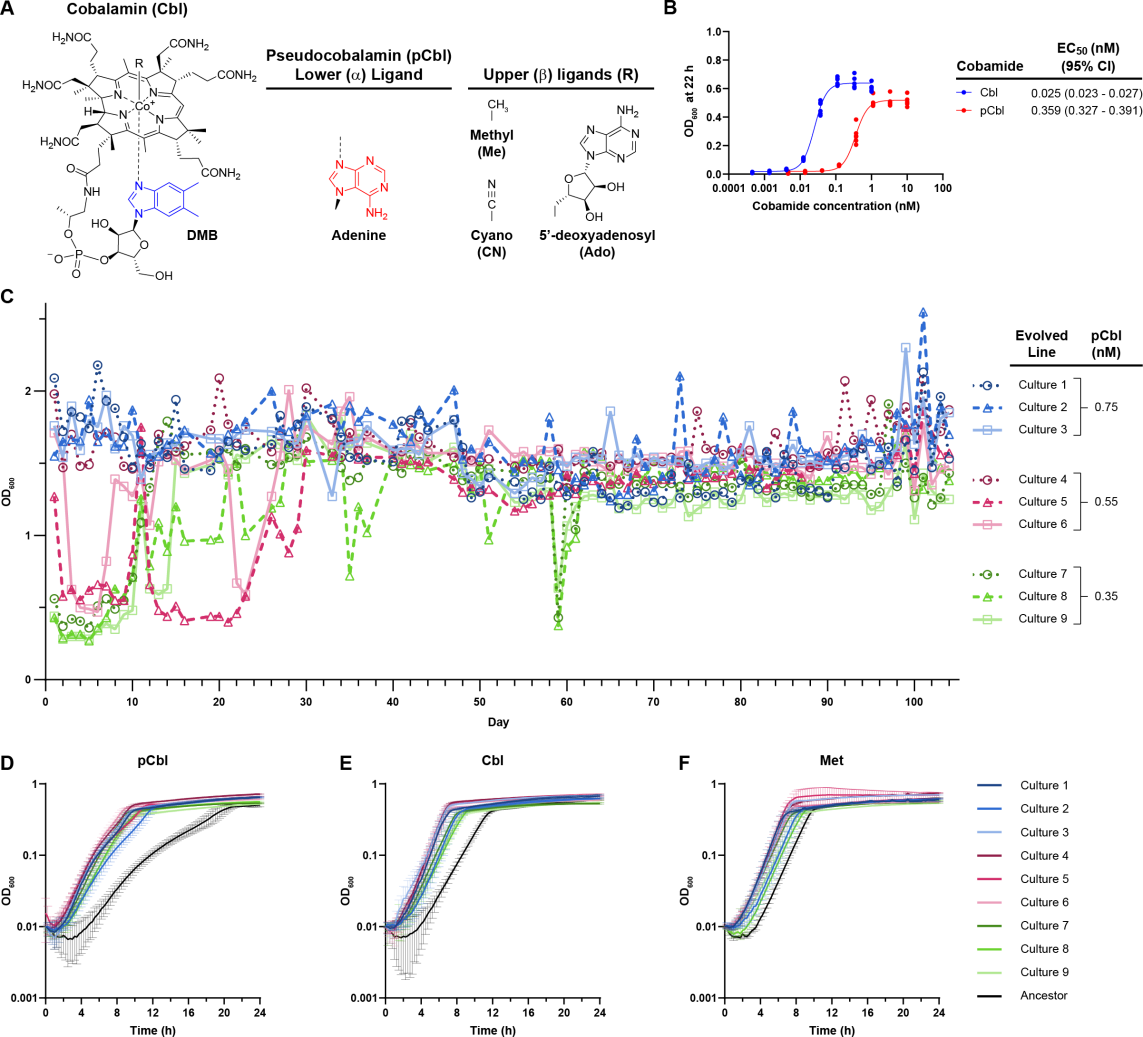
Laboratory evolution of *E. coli* improves its growth with pCbl. (**A**) Structure of cobalamin (Cbl; **B_12_**) with its lower ligand 5,6-dimethylbenzimidazole (DMB) in blue. Pseudocobalamin (pCbl) contains adenine (red) as its lower ligand. Cobamide upper ligands characterized in this study are shown. (**B**) Dose–response curves of *E. coli* ∆*metE* grown in the absence of methionine with various concentrations of Cbl or pCbl. OD_600_ was recorded after 22 hours. EC_50_ values and 95% confidence intervals of six biological replicates for each cobamide are shown. (**C**) Growth of *E. coli* ∆*metE* cultures during laboratory evolution. Three biological replicate cultures of *E. coli* ∆*metE* were passaged daily in M9 medium supplemented with 0.75, 0.55, or 0.35 nM pCbl for 104 days. OD_600_ was measured prior to diluting 1:100 into fresh medium every 24 hours. (**D–F**) Growth curves of evolved populations (Day 104) and the ancestral ∆*metE* strain in media with 0.35 nM pCbl (**D**), 0.35 nM Cbl (**E**), or 0.1 mg/mL Met (**F**). The average of three biological replicates is shown for panels D–F; error bars represent standard deviation.

Cobamides, the vitamin B_12_ family of metabolites, are used by most bacteria as cofactors for diverse metabolic processes including carbon metabolism, synthesis of methionine and deoxyribonucleotides, and natural product biosynthesis (1). They are produced exclusively by prokaryotes, although most bacteria that use cobamides are incapable of *de novo* synthesis (2, 3). Cobamides are modified tetrapyrroles (corrinoids) with a central cobalt ion that can coordinate to variable upper and lower axial ligands (2). The upper (β) ligand varies depending on catalytic function: a 5ʹ-deoxyadenosyl group is used for radical-based molecular rearrangements, while a methyl group is used for methyltransfer reactions (Fig. 1A). While B_12_ (cobalamin, Cbl) (Fig. 1A) is the best-studied cobamide due to its importance in human health (4), nearly 20 other cobamides with structural variability in the lower (α) axial ligand have been described (5–7). Different cobamides have distinct effects on microbial growth because cobamide-dependent growth and enzymatic function are differentially impacted by the lower ligand structure (6, 8–15). Diverse assortments of cobamides have been found in microbial communities from host-associated and environmental sources, and variability in cobamide abundances has been observed even in samples derived from similar sources (5, 16, 17). Recent studies have shown that microbial community composition can be significantly altered by addition of certain cobamides, suggesting cobamides can influence microbiomes (18–22). Because microbes can be exposed to different cobamides as their environments shift, they may encounter cobamides that function at varying levels of effectiveness for their metabolism. Given that cobamide structure and availability impact bacterial fitness and community structure, it is important to understand how bacteria are genetically wired to deal with different cobamides.

Many organisms have evolved strategies to cope with the absence of preferred cobamides. Certain bacteria, archaea, and algae carry out cobamide remodeling, whereby nonpreferred cobamides are converted into forms that can be used by their cobamide-dependent enzymes (9, 23–27). In addition, many bacteria encode cobamide-independent alternative enzymes or pathways, circumventing the need for cobamides for certain processes (3, 28). For example, cobamide-independent methionine synthase (MetE) and ribonucleotide reductases are each found in over half of bacterial genomes, including in approximately 80% and 40% of genomes that encode cobamide-dependent counterparts to these enzymes, respectively (3). Bacteria can also tailor their genetic response to the cobamides they prefer via selectivity in riboswitches, noncoding RNA elements in the 5ʹ untranslated region (UTR) of mRNA that, upon binding to specific cobamides, typically downregulate the expression of cobamide biosynthesis enzymes, transporters, and cobamide-independent enzymes (29–32).

Here, we carried out a laboratory evolution experiment in *Escherichia coli* to investigate whether there are additional genetic strategies that microbes may employ to improve their use of less-preferred cobamides. Due to its short generation time and genetic tractability, *E. coli* has been used extensively to address a range of biological questions via laboratory evolution, followed by genetic analysis. Although *E. coli* does not require exogenous cobamides in the absence of methionine because it contains *metE*, a Δ*metE* mutant relies on the cobamide-dependent methionine synthase MetH. Adeninylcobamide (pseudocobalamin, pCbl) (Fig. 1A) is a cobamide present in diverse environments, such as the human gut (16), but we find it is used less effectively compared to Cbl by *E. coli*. In our evolution experiment, we found that, indeed, an *E. coli* ∆*metE* mutant can improve its growth with pCbl via several genetic strategies. Different sets of mutations were found in evolved lines provided with different pCbl concentrations, but a common strategy that emerged was increasing the expression of the gene encoding the outer membrane corrinoid transporter BtuB by 300-fold, which provided a competitive advantage in limiting concentrations of cobamides. We additionally found that evolved lines and engineered strains that overexpress the corrinoid adenosyltransferase BtuR are better adapted for growth on pCbl. As a result, this study has revealed a previously unknown role for BtuR in MetH-dependent growth.

## RESULTS

### Laboratory evolution of *E. coli* improves the use of pCbl during MetH-dependent growth

The *E. coli* MG1655 ∆*metE* strain, which requires MetH activity for growth in minimal medium lacking methionine, prefers Cbl over pCbl, as revealed by comparing growth with the two cobamides (Fig. 1B). Specifically, the concentration of pCbl necessary for half-maximal growth (EC_50_) of this strain is over tenfold higher than for Cbl, and the maximal growth yield (OD_600_) is lower with pCbl (Fig. 1B). Although *E. coli* encodes three other cobamide-dependent enzymes, methylmalonyl-CoA mutase (ScpA/MCM), ethanolamine ammonia-lyase (EAL), and epoxyqueuosine reductase (QueG), they are unlikely to affect this phenotype. *scpA* has almost no expression in M9 medium (33), while work in the closely related species *Salmonella enterica* has shown that EAL expression is induced only in the presence of both AdoCbl and ethanolamine (34). Furthermore, deletion of *queG* does not alter the growth of the ∆*metE* strain with Cbl or pCbl (Fig. S1).

We therefore used the ∆*metE* strain to perform a laboratory evolution experiment with pCbl to determine whether *E. coli* can improve its use of this less-preferred cobamide. Nine independent cultures of the *E. coli* ∆*metE* strain were passaged daily for 104 days for a total of approximately 700 generations in M9 minimal medium containing either 0.75, 0.55, or 0.35 nM pCbl (Fig. 1C). These concentrations were chosen because they encompass nearly saturating to limiting growth of the ancestral strain (Fig. 1B). Five of the nine cultures reached a maximal OD_600_ below 0.6 during some or all of the first 10 days, but exceeded an OD_600_ of 1.2 for nearly all passages after day 30, indicating they had adapted to the limiting pCbl conditions (Fig. 1C). When compared to the ancestor, all nine populations showed improved growth with 0.35 nM pCbl (Fig. 1D). The nine populations also showed improved growth with Cbl (Fig. 1E), suggesting that they had evolved better use of cobamides in general. Growth with Met was modestly improved in the evolved populations, indicating they had adapted to other features of the growth medium (Fig. 1F).

### Mutants in one evolved population have a growth advantage specifically with pCbl

**Fig 2:**
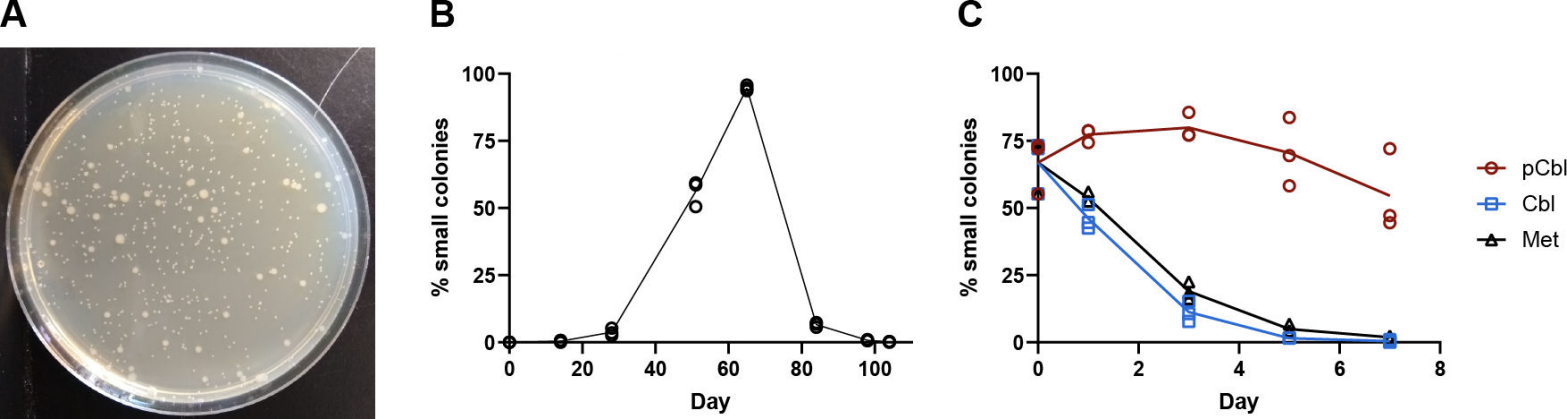
Small-colony variants emerge during evolution of Culture 8. (**A**) Plating of a population of Culture 8 on LB agar shows the regular and small-colony phenotypes. (**B**) The percentage of small-sized colonies was determined for archived populations of Culture 8. The Δ*metE* ancestor was used for the zero time point. (**C**) The percentage of small colonies is plotted for the day 65 archived population of Culture 8 grown with 0.35 nM pCbl, 0.35 nM Cbl, or 0.1 mg/mL Met over 7 days with daily passaging. Lines connect the means of three biological replicates.

We noticed that, when plated on LB agar, which contains methionine, some of the colonies from a passaged culture containing 0.35 nM pCbl (Culture 8) were distinctly smaller than the others (Fig. 2A). These small-colony variants first appeared on day 28, and they made up nearly the whole population on day 65 before being almost entirely lost by day 84 (Fig. 2B). Small-colony variants can arise during laboratory evolution experiments and often are not related to the phenotype being studied, as is the case in this study (see below) (35). Nonetheless, we took advantage of this distinct morphology by using it as a convenient markerless phenotype for further characterizing the pCbl-specific growth advantage of these strains.

Growth experiments on the archived culture from day 65 showed that the small-colony variants persisted following 1 week of daily passaging in the presence of pCbl, whereas their abundance decreased to 0 in media containing Cbl or Met (Fig. 2C). The percentage of small colonies in the cultures supplemented with pCbl started to decrease on day 5 of the experiment (Fig. 2C), suggesting that the same population dynamics may have been occurring that were seen during the laboratory evolution (Fig. 2B).

**Fig 3:**
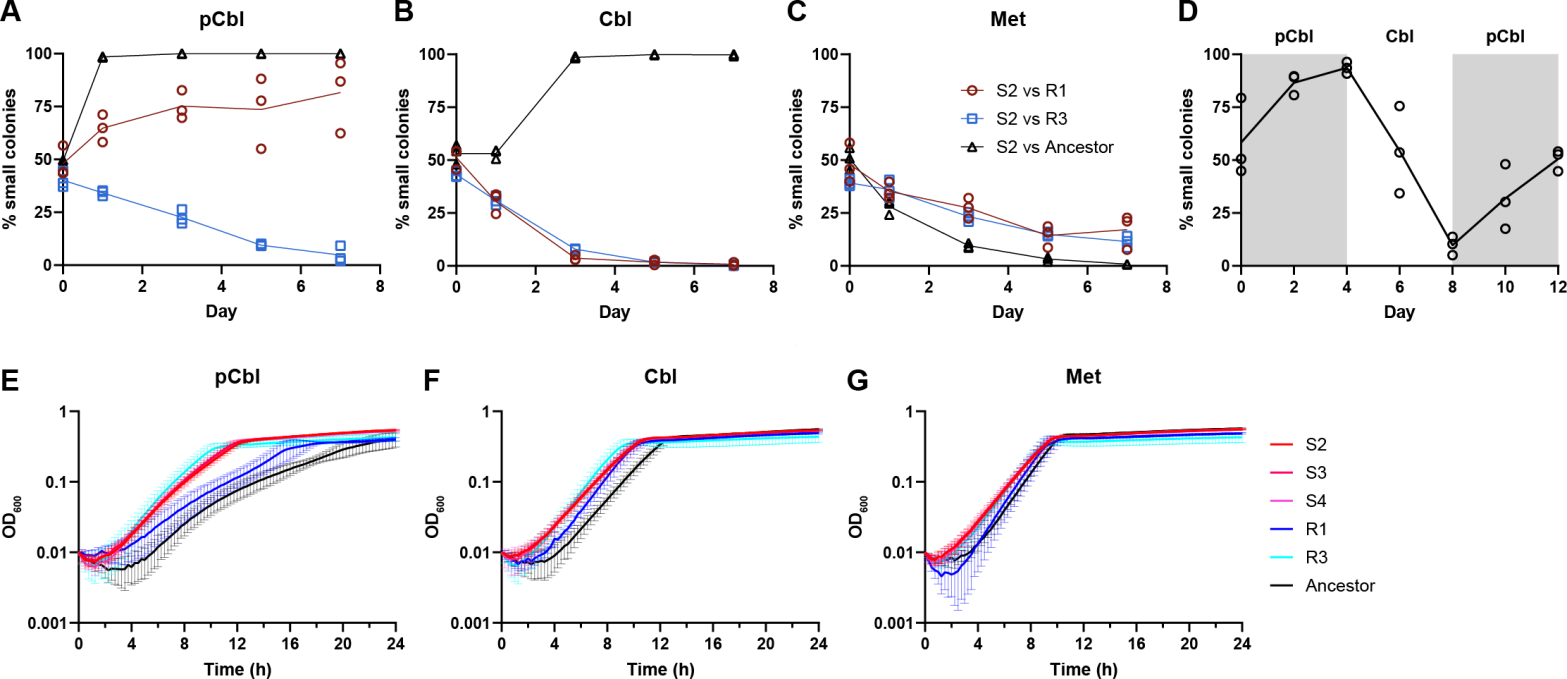
Growth characteristics of isolates S2, S3, S4, R1, and R3 from Culture 8. (**A–C**) Isolate S2 was competed against isolates R1 and R3 and the ancestor strain for 7 days with daily passaging in medium containing 0.35 nM pCbl (**A**), 0.35 nM Cbl (**B**), or 0.1 mg/mL Met (**C**). Cultures were diluted and plated on the indicated days to quantify the fraction of small colonies, corresponding to S2 strain abundance. (**D**) Isolates S2 and R1 were competed for 12 days with daily passaging in medium containing either 0.35 nM pCbl (shaded) or 0.35 nM Cbl (unshaded), and the fraction of small colonies (**S2**) was calculated as in panels A–C. Lines in A–D connect the means of three biological replicates. (**E–G**) Growth curves of isolates S2, S3, S4, R1, R3, and the ancestor strain in media containing 0.35 nM pCbl (**E**), 0.35 nM Cbl (**F**), or 0.1 mg/mL Met (**G**). The average of three biological replicates is shown; error bars represent standard deviation.

We isolated colonies with different sizes from the day 65 population and individually competed three “small” isolates (S2, S3, and S4) against two “regular” isolates (R1 and R3), as well as the ancestral ∆*metE* strain, in media containing either pCbl, Cbl, or Met. All three small isolates had similar phenotypes. When co-cultured, the small isolates outcompeted the ancestor in the presence of cobamides, taking over the entire population after a single passage with pCbl and after three passages with Cbl (Fig. 3A and B; Fig. S2A, B, D and E). In media with methionine, however, the ancestral strain outcompeted the evolved isolates (Fig. 3C; Fig. S2C and F). When the small isolates were competed against the two regular isolates, the small isolates were outcompeted when grown with Cbl and Met, but we observed contrasting phenotypes with pCbl (Fig. 3A through C; Fig. S2). The small isolates had a competitive advantage over regular isolate R1 but were outcompeted by R3 (Fig. 3A; Fig. S2A and D).

The competitive advantage of the small isolates with pCbl was further confirmed by co-culturing isolates S2 and R1 in medium supplemented alternately with pCbl and Cbl. When passaged with pCbl for 4 days, the proportion of S2 increased to over 90%, but after switching to the Cbl-containing medium, the proportion of S2 decreased to less than 10% after 4 days. A subsequent return to pCbl resulted in an increase in S2 (Fig. 3D).

Growth of the isolates in pure culture was largely consistent with their competition phenotypes in co-culture. Isolates S2, S3, and S4, which outcompeted the ancestor and isolate R1 in media with pCbl, grew faster with shorter lag times in pure culture with pCbl (Fig. 3E; Fig. S3A and B). In medium with Cbl, in which S2, S3, and S4 outcompeted the ancestor in co-culture, the small isolates had shorter lag times and growth rates similar to those of the ancestor (Fig. 3F; Fig. S3C and D). Isolate R3, which outcompeted the small isolates in all three media conditions, had slightly higher growth rates with similar lag times to the small isolates in each medium (Fig. 3E through G; Fig. S3). Finally, isolates S2, S3, and S4 were outcompeted by both the ancestor and isolate R1 in co-culture with Met, as well as by R1 with Cbl, despite having shorter lag times than these strains in pure culture, perhaps due to their slightly lower growth rates (Fig. 3F and G; Fig. S3C through F). Taken together, these results suggest that all of the evolved isolates have acquired one or more mutations that confer a growth advantage with cobamides. Furthermore, based on the growth phenotypes of strains S2, S3, S4, and R3 with pCbl, these strains likely have one or more mutations that confer a specific advantage with pCbl.

### Evolved isolates have mutations that increase the expression of cobamide-related genes

**Fig 4:**
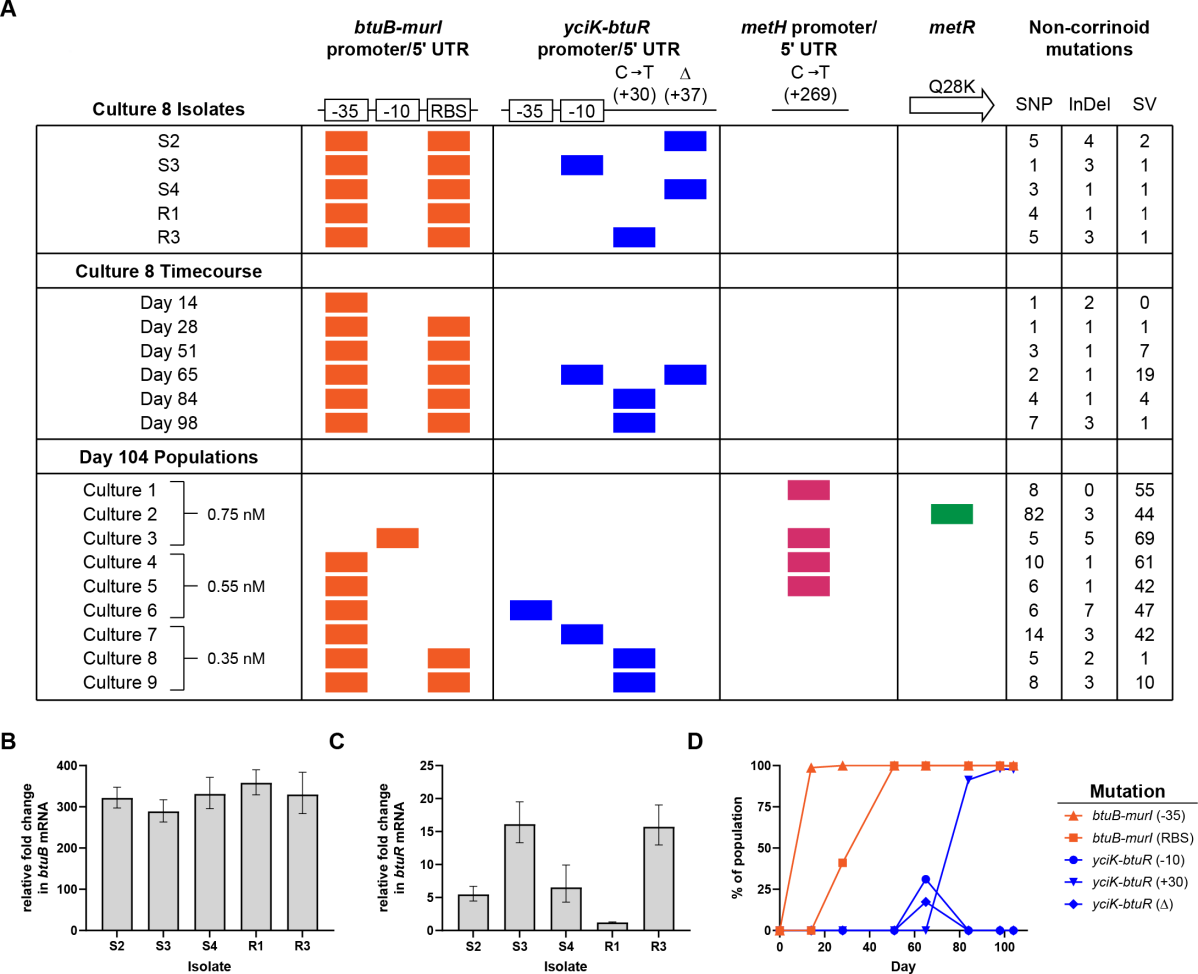
Corrinoid-related genes are mutated and upregulated during evolution. (**A**) Colored boxes show the presence of the indicated mutations affecting corrinoid-related genes in isolates and archived time points from Culture 8 and in the endpoint (Day 104) populations of all evolved lines. The promoter for the *yciK-btuR* operon has not been experimentally characterized and was predicted by PromoterHunter (37). The specific changes in the promoters and 5ʹ UTRs of *btuB-murI* and *yciK-btuR* are shown in Fig. S4. *yciK-btuR* is on the minus strand of the chromosome, and the +30 change (C to T) is the reverse complement of the mutation in the 5ʹ UTR. The concentration of pCbl present during the evolution of each line (Cultures 1–9) is indicated. The numbers of SNPs, InDels, and SVs affecting non-corrinoid-related genes in each sequenced isolate or population are shown (see Table S1 for list of genes and mutations). (**B and C**) *btuB* (**B**) and *btuR* (**C**) transcript levels in evolved isolates relative to the Δ*metE* ancestor from cultures containing 1 nM pCbl, as determined by RT-qPCR. Mean and error were calculated from three biological replicates. (**D**) Frequency of the *btuB-murI* and *yciK-btuR* mutations within the Culture 8 population during the evolution experiment.

To identify the mutations acquired during the evolution experiment, we performed whole-genome sequencing on the isolates from Culture 8. Each isolate has a unique set of mutations, which range from 8 to 14 single-nucleotide polymorphisms (SNPs), insertions and deletions (InDels), or structural variants (SVs) (Fig. 4A; Table S1). This includes mutations in known cobamide-related genes in all five isolates. They each have the same two SNPs in the promoter and ribosome-binding site (RBS) of the *btuB-murI* operon, which encodes the outer membrane corrinoid transporter BtuB and the glutamate racemase MurI (Fig. 4A). We predict that these mutations result in increased expression of the operon (see Fig. S4A for details). RT-qPCR on the ancestor strain and the evolved isolates confirmed that the operon is overexpressed in the evolved isolates. *btuB-murI* transcript levels in the evolved isolates were approximately 300-fold higher than in the ancestor (Fig. 4B), which could be due to a combination of both increased transcription of the operon and disruption of riboswitch-mediated repression (Fig. S4A) (36).

In addition to the mutations in *btuB-murI*, all of the isolates, except R1, have a mutation in the predicted promoter or 5ʹ UTR of the *yciK-btuR* operon (Fig. 4A; Fig. S4B). *yciK* is an uncharacterized gene annotated as a putative oxidoreductase, and *btuR* encodes an adenosyltransferase that installs a 5ʹ-deoxyadenosyl group as the β ligand of corrinoids. RT-qPCR analysis of the evolved isolates showed that isolates with *btuR* mutations (S2, S3, S4, and R3) had fivefold to 15-fold higher levels of *yciK-btuR* mRNA compared to the ancestor, while mRNA levels similar to the ancestor were observed in isolate R1, which does not have a mutation in *yciK-btuR* (Fig. 4C). These results suggest that growth of *E. coli* with limiting amounts of pCbl can be improved by increasing the expression of these two operons.

Sequencing of the archived populations of Culture 8 enabled us to follow the emergence of these mutations during the evolution experiment (Fig. 4A and D; Table S1). Both *btuB-murI* mutations arose early in the time course and were retained within the entire population by day 51 (Fig. 4D). Notably, mutations in the −35 element and RBS were present by days 14 and 28, respectively, coinciding with increases in the OD_600_ of the culture (Fig. 1C).

The two *yciK-btuR* mutations found in the small isolates were first detected on day 65, in 31% and 17% of the population, respectively (Fig. 4D). At all of the following time points, however, only the C to T mutation found in isolate R3 was detected in the population. Given that isolate R3 outcompeted isolates S2, S3, and S4 in pCbl (Fig. 3A; Fig. S2A and D), it is likely that descendants of R3 became dominant in the population after day 65. An *rpsK* mutant was observed to have a small-colony phenotype in a previous study in *E. coli* (38), and we found here that the frequency of an *rpsK* mutation correlated with the prevalence of small colonies in the population (Table S1).

### *E. coli* adapts differently with limiting versus near-saturating pCbl

The endpoint (Day 104) archives of the nine evolved cultures were also sequenced. The populations have several of the cobamide-associated mutations found in the Culture 8 isolates and archives, as well as mutations in nearly 400 additional genes, including 75 genes found mutated in more than one sample (Fig. 4A; Table S1). In each population, at least one known cobamide-related gene is mutated, with different genes affected depending on the concentration of pCbl present during the evolution (Fig. 4A). The two other populations passaged with 0.35 nM pCbl developed the same mutations upstream of *btuB-murI* and *yciK-btuR* as those found in Culture 8 (Fig. 4A, Cultures 7 and 9). In contrast, only a minor fraction (14%) of one population passaged with 0.75 nM pCbl has a mutation upstream of *btuB-murI*, and one population passaged with 0.55 nM pCbl has a mutation upstream of *yciK-btuR* (Cultures 3 and 6, respectively; Fig. 4A; Table S1), although all three populations grown with 0.55 nM pCbl have the same *btuB-murI* promoter mutation found in the isolates.

All of the evolved populations lacking mutations in *yciK-btuR* have a mutation upstream of *metH* or in the coding sequence of *metR*, a transcriptional activator of *metH* (39), in 50% or more of the population (Cultures 1–5; Fig. 4A; Table S1). It is unclear how these mutations affect *metH* expression; the *metH* mutation is not located in the promoter, RBS, or MetR-binding site (40), while the *metR* mutation is located in its DNA-binding domain (41). Taken together, these results suggest that increasing cobamide uptake and adenosylation are effective strategies for improving growth with limiting-to-moderate pCbl concentrations, while changing the expression of *metH* facilitates adaptation at higher concentrations of pCbl.

### Overexpression of the corrinoid uptake gene *btuB* is advantageous at limiting cobamide concentrations

**Fig 5:**
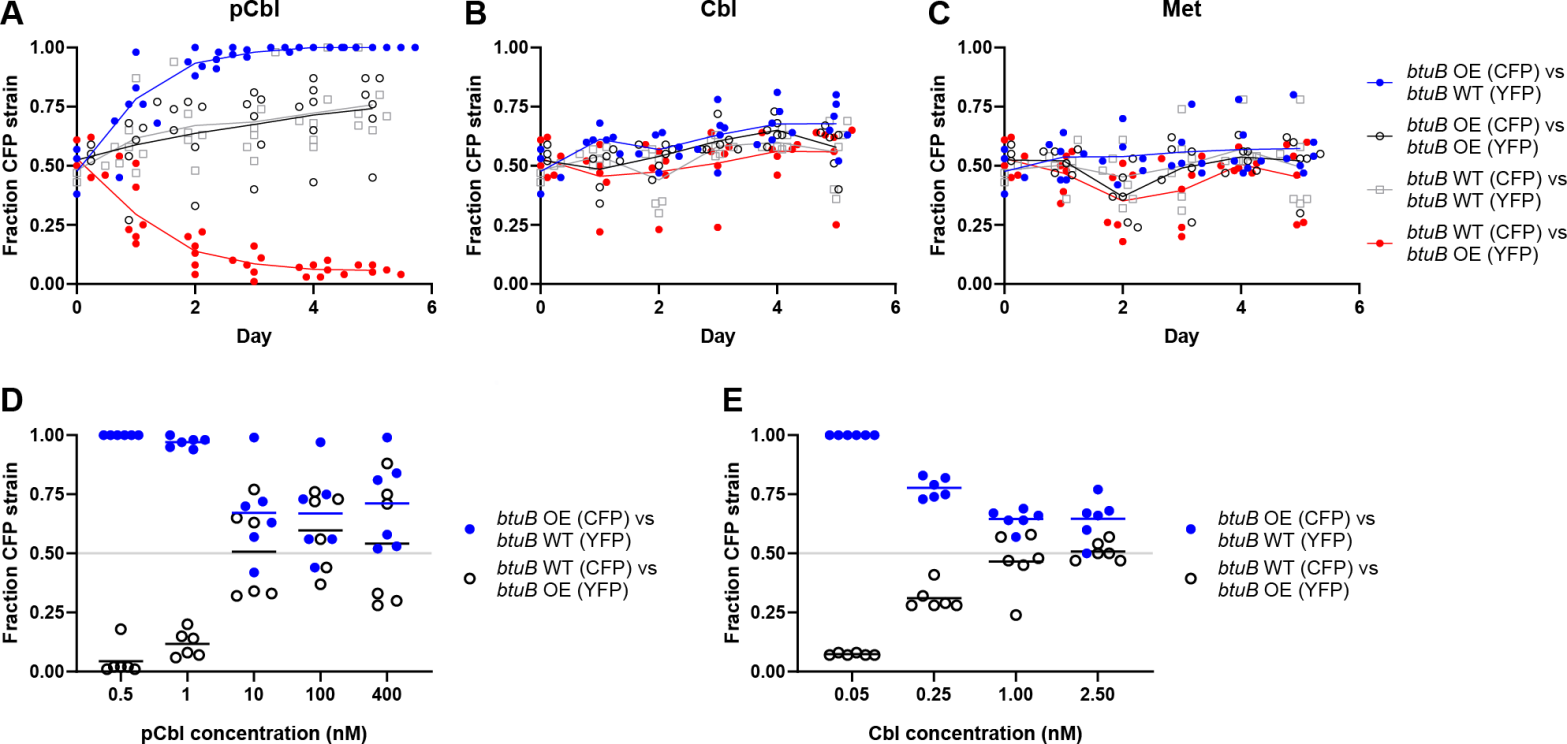
Overexpression of *btuB* confers a competitive advantage at limiting cobamide concentrations. (**A–C**) *E. coli* ∆*metE* strains overexpressing *btuB* (OE) or producing native levels of *btuB* (WT), each expressing either CFP or YFP, were competed in co-culture for 5 days with daily passaging in medium containing either 1 nM pCbl (**A**), 1 nM Cbl (**B**), or 0.1 mg/mL Met (**C**). The fraction of the CFP-expressing strain in each co-culture is plotted. Control co-cultures containing CFP- and YFP-expressing strains with the same genetic background (black and gray) were included to rule out a growth disadvantage caused by either of the fluorescent proteins. (**D and E**) The CFP- and YFP-expressing strains that overexpress *btuB* (OE) or produce native levels of *btuB* (WT) were competed in co-culture at different concentrations of pCbl (**D**) or Cbl (**E**). Fluorescence was measured on day 3 following daily passaging. Lines represent the means of six biological replicates.

Having found that the operons containing *btuB* and *btuR* are upregulated in the evolved isolates, we sought to test whether overexpression of each gene separately affects cobamide-dependent growth. We first investigated the influence of *btuB* alone since the glutamate racemase MurI, encoded in the same operon, has no known function in cobamide metabolism (42). We constructed a strain with a Δ*metE* mutation that overexpresses *btuB* by inserting a second copy of the gene into the chromosome, with its promoter containing the G to T mutation found in the −35 element of the evolved isolates. This strain was competed against a Δ*metE* control strain (see *Methods*), with each strain expressing either CFP or YFP to monitor their abundances in co-culture. We found that overexpression of *btuB* conferred a competitive advantage in media containing 1 nM pCbl, but not 1 nM Cbl or 0.1 mg/mL Met (Fig. 5A through C). However, competing the strains in varying cobamide concentrations showed that *btuB* overexpression is beneficial with both pCbl and Cbl, but only at concentrations at which the cobamide is limiting, namely, 1 nM and less for pCbl, and under 0.25 nM for Cbl (Fig. 5D and E). Thus, the *btuB* mutations that arose during passaging with limiting pCbl presumably improved *E. coli*’s ability to import cobamides to support MetH-dependent growth.

### The corrinoid adenosyltransferase gene *btuR* is required for optimal MetH-dependent growth

**Fig 6:**
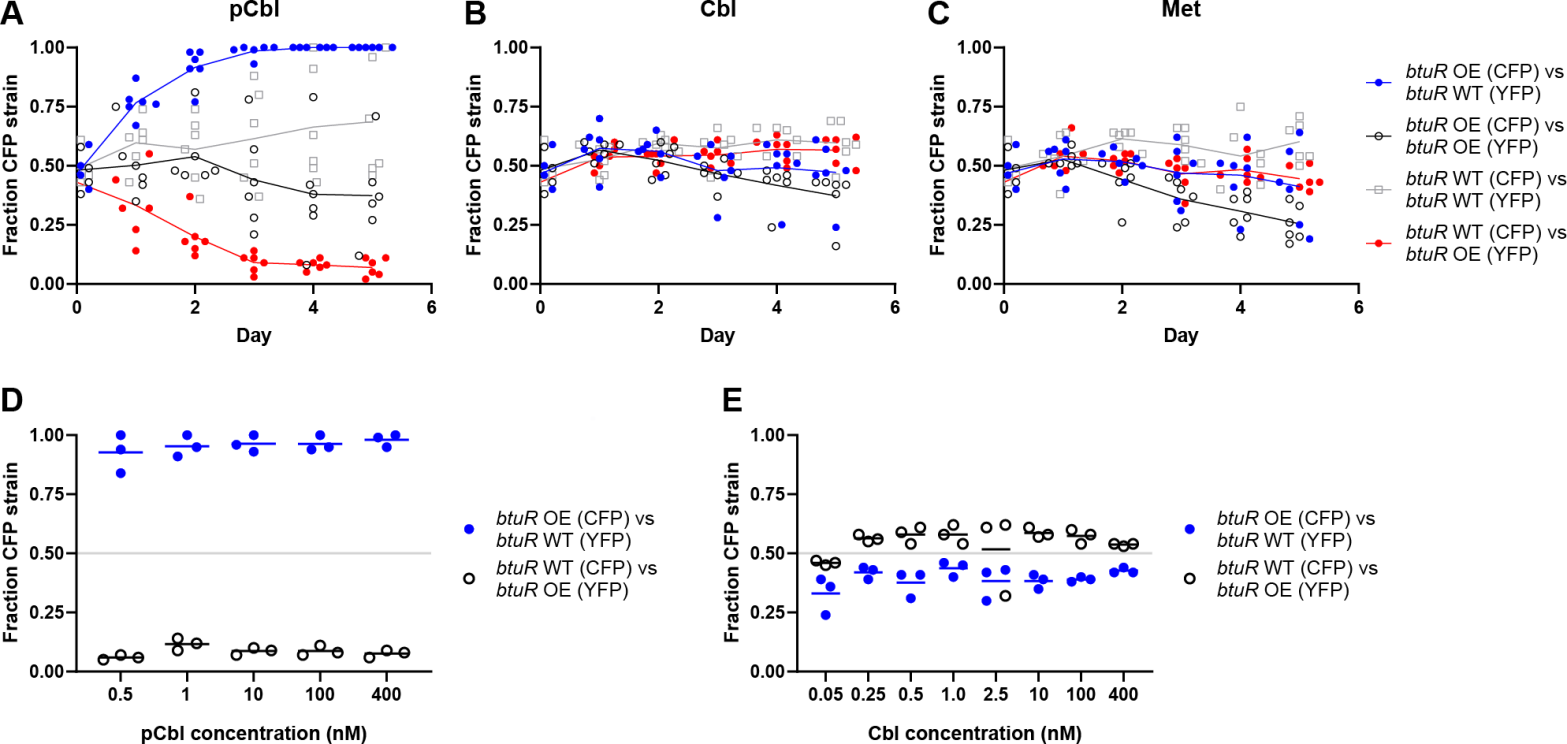
Overexpression of *btuR* confers a competitive advantage only during growth with pCbl. (**A–C**) *E. coli* ∆*metE* strains overexpressing *btuR* (OE) or producing native levels of *btuR* (WT), each expressing CFP or YFP, were competed in co-culture for 5 days with daily passaging in medium containing either 1 nM pCbl (**A**), 1 nM Cbl (**B**), or 0.1 mg/mL Met (**C**). The fraction of the CFP-expressing strain in each co-culture is plotted. Control co-cultures containing CFP- and YFP-expressing strains with the same genetic background (black and gray) were included to rule out a growth disadvantage caused by either of the fluorescent proteins. (**D and E**) The CFP- and YFP-expressing strains that overexpress *btuR* (OE) or produce native levels of *btuR* (WT) were competed in co-culture at different concentrations of pCbl (**D**) or Cbl (**E**). Fluorescence was measured on day 3 following daily passaging. Lines represent the means of three biological replicates.

Next, we assessed whether increasing *btuR* expression impacts MetH-dependent growth by overexpressing *btuR* on a plasmid. We found that, similar to the results with *btuB*, a strain overexpressing *btuR* outcompeted a strain with wild-type *btuR* levels when grown with pCbl, but not with Cbl or Met (Fig. 6A through C). Although it is in an operon with *yciK*, *btuR* alone was responsible for this phenotype as overexpression of *yciK* did not confer a growth advantage with pCbl and co-expression of *yciK* with *btuR* did not influence the effect of overexpression of *btuR* alone (Fig. S5). However, unlike *btuB*, overexpression of *btuR* conferred a competitive growth advantage even at concentrations of up to 400 nM pCbl, but failed to confer an advantage at any concentration of Cbl tested (Fig. 6D and E).

Cobamide riboswitches can be affected by the upper ligand of the cobamide (43, 44), so the phenotypes observed from an increase of BtuR could be due to altered regulation of *btuB* expression via its riboswitch. However, this would require the repressing *btuB* riboswitch to become less responsive to pCbl upon adenosylation, as increased expression of *btuB* is beneficial for growth at low concentrations of pCbl (Fig. 5D). Instead, adenosylated cobamides were found to have high binding affinity for this riboswitch (45). Furthermore, given that *btuB* overexpression results in competition phenotypes distinct from *btuR* overexpression with both pCbl and Cbl (compare Fig. 5D, E, 6D and E), it is unlikely that the BtuR-associated phenotype is due to an effect on *btuB* expression. Nevertheless, we examined the *btuR* overexpression phenotype in a strain that also overexpresses *btuB* and found that *btuB* overexpression does not alter the *btuR* phenotype (Fig. S6).

**Fig 7:**
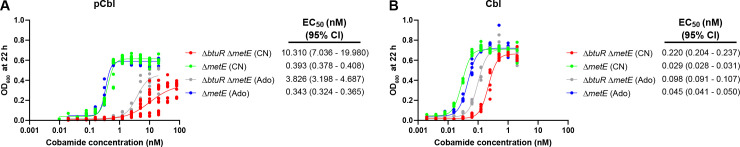
Deletion of *btuR* causes poorer growth with pCbl and Cbl. (**A and B**) Cobamide dose–response curves are shown for *E. coli* ∆*btuR* ∆*metE* and ∆*metE* strains grown with various concentrations of CNpCbl and AdopCbl (**A**) or CNCbl and AdoCbl (**B**) with no added methionine. OD_600_ was recorded after 22 hours of growth. EC_50_ values and 95% confidence intervals were calculated from six to 18 biological replicates for each cobamide.

In the ∆*metE* mutant, cobamides are used by the MetH enzyme to transfer methyl groups from methyltetrahydrofolate to homocysteine by alternately methylating and demethylating the cobamide at the β position. It was, therefore, puzzling to find that overexpression of BtuR, which adenosylates cobamides at the β position, improves MetH-dependent growth. To further explore the role of BtuR in MetH-dependent growth, we deleted *btuR* and performed growth assays with pCbl or Cbl with either cyano (CN, as in Fig. 1 to 6) or adenosyl (Ado) β ligands (Fig. 1A). Growth measurements with these cobamides showed that a ∆*btuR* ∆*metE* strain has impaired growth in cyanopseudocobalamin (CNpCbl), with a lower maximum OD_600_ and an EC_50_ over 25-fold higher than the ∆*metE* strain (Fig. 7A). Growth with adenosylpseudocobalamin (AdopCbl) led to a higher maximum OD_600_ and lower EC_50_ of the ∆*btuR* ∆*metE* strain, although growth was still considerably impaired compared to that of the ∆*metE* strain (Fig. 7A). A similar trend was observed when these strains were cultured with cyanated versus adenosylated forms of Cbl (CNCbl and AdoCbl, respectively), although the growth impairment of the ∆*btuR* ∆*metE* strain was more modest (Fig. 7B). Together, these results confirm that *btuR* influences MetH-dependent growth in *E. coli*.

To examine how BtuR may be affecting MetH function, we assessed whether *btuR* expression levels affect the amount of methylcobamide present in the cell. As methylcobamide is only generated and subsequently used during each catalytic cycle, a change in methylcobamide levels suggests a change in cobamide binding, activity, or abundance of MetH. We incubated 250 pmol of CNpCbl or CNCbl with *E. coli* strains expressing native levels of *btuR* (*btuR* WT), overexpressing *btuR* (*btuR* OE), or expressing no *btuR* (∆*btuR*) for 6 hours and then analyzed the methylcobamide and adenosylcobamide contents within the cell (Fig. S7). We found that for both pCbl and Cbl, overexpression of *btuR* did not alter the methylcobamide or adenosylcobamide levels as compared to native *btuR* expression. This suggests that the competitive advantage provided by *btuR* overexpression during growth with pCbl is not due to an increase in MetH activity or adenosylcobamide levels. Studies to resolve the unexpected role of BtuR during *E. coli* MetH-dependent growth are ongoing.

## DISCUSSION

Cobamides are considered key shared nutrients because they function as cofactors for numerous microbial processes but are only produced by a subset of prokaryotes. They have been detected in diverse microbial communities, both environmental and host-associated, and a wide range in cobamide levels has been observed across ecosystems, with some dominated by one or two cobamides, while others contain up to eight different types (16). These differences in cobamide diversity across environments are noteworthy in light of the observation that many bacteria have preferences for particular cobamides and most bacteria rely on cobamides produced by other microbes. This raises the question of how bacteria adapt in the presence of nonpreferred cobamides. We addressed this question by using a cobamide-dependent mutant of *E. coli* in a laboratory evolution experiment. We found that *E. coli* is, indeed, capable of improving its growth with pCbl via genetic changes, and it uses differing strategies depending on the availability of the nutrient. Competition experiments and genetic analyses revealed regulation of corrinoid uptake as a limiting factor in *E. coli* and a previously unappreciated role for the corrinoid adenosyltransferase BtuR in MetH-dependent growth.

It is noteworthy that as a native of the human gut, *E. coli* prefers Cbl to pCbl, as the latter was the third-most abundant corrinoid found in human fecal samples, while Cbl constituted only a minor fraction (5). While it is not known what underlies *E. coli*’s preference for Cbl during MetH-dependent growth, studies in other organisms have found that cobamide-dependent enzymes can have varying activities with and binding affinities for different cobamides. We previously reported that homologs of MCM have different binding affinities for different cobamides and that these cobamide-binding affinities largely mirror MCM-dependent growth with different cobamides in *Sinorhizobium meliloti* (13). Other studies have shown that MetH orthologs from different organisms display distinct preferences for different cobamides (9, 14, 25). With *E. coli*, we hypothesized that passaging with pCbl would lead to the accumulation of mutations in the MetH enzyme that improved its ability to bind and use pCbl. Though we observed mutations that presumably impact *metH* expression, no mutations were found in the *metH* coding sequence. Altering the expression of corrinoid-related genes was the general outcome of our evolution experiment, suggesting that modifying the regulation of cobamide metabolism may be a more readily accessible mechanism of adaptation than changes to the specificity of the dependent enzyme, particularly in our experimental timeframe. Changes to gene expressions are routinely observed in laboratory evolution experiments, including the targeting of global regulators (46, 47).

While mutations in *metH* and its transcriptional activator *metR* were found at the higher concentration of pCbl, mutations upregulating the outer membrane corrinoid transporter BtuB arose primarily when pCbl was found in a limiting concentration, consistent with its role in corrinoid uptake. Indeed, we previously showed that overexpression of the corrinoid transport machinery in *Bacillus subtilis* increases the amount of cobamide imported (32). Although it is not known whether the affinity of BtuB for Cbl versus pCbl differs, structural and biochemical studies with Cbl and a corrinoid intermediate lacking a lower ligand (cobinamide) have shown that the lower ligand is of minimal importance to binding, with BtuB primarily interacting with the corrin moiety (48–50). Instead, greater *btuB* expression, leading to increased pCbl uptake, could compensate for decreased efficiency with pCbl at a later step such as adenosylation by BtuR or utilization by MetH. The evolved mutations upregulating *btuB* may also be a consequence of the selective pressure from competing for limiting pCbl. Cobamide uptake is critical for colonization by the human gut commensal bacterium *Bacteroides thetaiotaomicron* in a mouse model (51), and *B. thetaiotaomicron* and other *Bacteroides* species encode multiple corrinoid transport systems, which include high-affinity corrinoid binding proteins absent from *E. coli* (52). These uptake systems are thought to enable *Bacteroides* to outcompete other microbes for corrinoids, allowing for successful gut colonization (53, 54). Our observation that corrinoid uptake and competitiveness in *E. coli* can readily be improved via mutations in the *btuB* promoter or RBS suggests that *E. coli* is not evolved to maximize corrinoid uptake, despite it being a member of the gut microbiota like *B. thetaiotaomicron. E. coli* could be under less selective pressure to maximize corrinoid uptake because, unlike *B. thetaiotaomicron*, *E. coli* has the cobamide-independent methionine synthase *metE* as well as *metH*, rendering it less dependent on exogenous cobamides. In addition, increased expression of *btuB* may not always be beneficial in natural settings. It could negatively impact outer membrane function or, as a phage receptor (55), increase its susceptibility to phage infection.

The BtuR corrinoid adenosyltransferase is responsible for installing a 5ʹ-deoxyadenosine moiety as the β ligand of cobamides to produce adenosylcobamides (56), which are required for the subset of cobamide-dependent enzymes, such as MCM, that carry out radical-based reactions using a 5ʹ-deoxyadenosyl radical (57). However, no role for adenosylcobamides has been proposed for methyltransferases such as MetH, which use cobamides without a β ligand to shuttle methyl groups from a methyl donor to a substrate, transiently generating methylcobamide during each catalytic cycle. Therefore, it was surprising to find that, in MetH-dependent *E. coli*, overexpression of *btuR* provides a competitive advantage during growth with pCbl, deletion of *btuR* impairs growth with both pCbl and Cbl, and supplementation of the Δ*btuR* mutant with adenosylcobamides only partially rescues the growth phenotype. These results suggest that adenosylcobamides, and perhaps the BtuR protein itself, could have previously unknown roles in MetH function. Some cobamide-dependent enzymes such as MCM require a corrinoid adenosyltransferase and other accessory proteins to load the cobamide cofactor into the enzyme (58–60). It is possible that BtuR fulfills such a role for MetH in *E. coli*, particularly for cobamides that function poorly as a cofactor for MetH, which may be the case for pCbl. Alternatively, adenosylcobamides and/or BtuR could facilitate SAM-dependent cobamide reactivation, a step required approximately every 2,000 turnovers for Cbl following spontaneous cofactor oxidation (61–63). Further studies of BtuR will be necessary to determine how this enzyme affects MetH function. In addition, while experiments with *E. coli* MetH have been unable to address the cofactor loading step and enzyme function with alternative cobamides because the protein is stable only when pre-loaded with Cbl during purification, the recent discoveries of MetH homologs that are stable in their apo forms may now enable such analyses (64, 65). Because pCbl functions more poorly than Cbl in *E. coli* MetH-dependent growth, our evolution experiment may have fortuitously uncovered a role for adenosylated cobamides in corrinoid-dependent physiology. Future work will be aimed at understanding the molecular mechanisms underlying these observations.

## MATERIALS AND METHODS

### Media and growth conditions

*E. coli* MG1655 ∆*metE* evolution was performed at 37°C with aeration in M9 glycerol (0.2%) minimal medium (66) with the indicated concentrations of pCbl. Cultures (20 mL) were grown in glass test tubes, with 0.2 mL transferred into fresh media every 24 hours. A sample of each population was archived on days 14, 28, 51, 65, 84, 98, and 104 in 25% glycerol and stored at −80°C. Before the start of the evolution experiment, the three replicate cultures were passaged daily for 16 days with a saturating level of pCbl (5 or 2.5 nM), while the appropriate concentrations for the evolution experiment were being determined.

M9 medium was supplemented with L-methionine (Met) at 0.1 mg/mL, unless indicated. Luria–Bertani (LB) agar was used as solid medium. For experiments with the *E. coli* ∆*metE* ancestor or evolved isolates from Culture 8, M9 medium was inoculated with individual colonies grown on LB agar. Pre-culturing of populations and strains in M9 medium was performed at 37°C with aeration.

### Strain construction

All strains used for evolution and mutant validation are derivatives of wild-type K12 strain MG1655. *E. coli* strains were cultured at 37°C with aeration in LB medium during strain construction. Media were supplemented with antibiotics at the following concentrations when necessary: kanamycin (kan), 25 mg/liter (pKIKO, pETmini); carbenicillin, 100 mg/liter (pCP20); chloramphenicol (Cm), 10–20 mg/liter (pACYCDuet-1). pKIKO*arsB*Km plasmids were propagated in *E. coli* DH5α containing λ*pir*.

The Δ*metE*::kan^R^ and Δ*btuR*::kan^R^ mutations from the Keio collection (67) were introduced by P1 transduction into *E. coli* strain MG1655 (68). The kanamycin resistance cassette was removed from Δ*metE*::kan^R^ by introducing the plasmid pCP20 as described, leaving the FRT site in place of the *metE* coding sequence (69).

An *E. coli* strain overexpressing *btuB* was created by integrating an additional copy of *btuB* at the *arsB* (arsenite transporter) locus using the KIKO system as described (70). pKIKO*arsB*Km was a gift from Lars Nielsen & Claudia Vickers (Addgene plasmid # 46766; http://n2t.net/addgene:46766; RRID:Addgene_46766). *E. coli btuB* with its promoter and riboswitch was cloned into pKIKO*arsB*Km, with the promoter containing the −35 element mutation (TTGACA) found in evolved populations and isolates. *btuB* also contained a synonymous mutation in codon 581 encoding a valine (GTT to GTA). The PCR-based method was used to integrate this construct at the *arsB* locus. The kanamycin resistance cassette alone from pKIKO*arsB*Km was integrated at the *arsB* locus to create a control strain. Both constructs were first integrated into MG1655 before being transduced via P1 into the Δ*metE* strain. Finally, the kanamycin resistance cassette was removed using pCP20. Strains were confirmed by PCR and Sanger sequencing. Plasmids pSG013 and pSG015, which contain mCerulean and mCitrine (abbreviated CFP and YFP in the text), respectively, were each transformed into the two strains to enable tracking based on fluorescence (71).

The *btuR* and *yciK* genes were overexpressed in a pACYCDuet-1 plasmid in which the T7 promoters were replaced with a *lac* promoter and operator (pACYCDuet-1-pLac). This enabled repression of gene expression in the presence of glucose (0.02% in LB, 0.2% in M9) and expression in the absence of glucose due to the leakiness of the *lac* promoter. *E. coli btuR*, *yciK*, and the *yciK-btuR* operon were each cloned downstream of the *lac* promoter in pACYCDuet-1-pLac. mCerulean and mCitrine genes from pSG013 and pSG015 (with J23100 promoter and B0034 RBS) were inserted between the chloramphenicol resistance cassette and p15A origin in each of these plasmid constructs to enable tracking of strains by fluorescence measurements (71).

### Cobamide reagents

CNCbl, AdoCbl, and MeCbl were purchased from MilliporeSigma. CNpCbl was extracted from *Propionibacterium acidi-propionici* strain DSM 20273 and HPLC-purified as described (72, 73). We previously showed that HPLC purification of CNCbl from a bacterial culture did not affect its performance (74). AdopCbl was chemically synthesized from CNpCbl and purified as described (13). Cobamides were quantified spectrophotometrically (13, 26). Cbl and pCbl were used in their cyano forms (CNCbl and CNpCbl), unless otherwise indicated.

### Growth assays and competition experiments

To quantify the percentage of small colonies present during the evolution of Culture 8, archived populations were cultured overnight in M9 glycerol medium supplemented with 0.35 nM pCbl, diluted, and plated on LB agar. The colony size phenotype was judged visually, with the largest colonies considered to have a “regular” colony size phenotype and those distinctly smaller having a “small” colony phenotype.

Growth assays and competition experiments were performed in 200-µL cultures in 96-well plates (Corning, 3598). For growth curves, populations or isolates were pre-cultured in M9 glycerol supplemented with 0.35 nM pCbl, while cultures for cobamide dose–response assays were supplemented with Met. Cells from saturated cultures were collected by centrifugation, resuspended in M9 glycerol, and OD_600_ was measured. Each culture was then inoculated at a starting OD_600_ of 0.01 in M9 glycerol medium with the indicated supplement. Then, 96-well plates were sealed with Breathe-Easy (Diversified Biotech). Growth assays were performed in a BioTek Synergy 2 microplate reader with shaking at medium speed at 37°C and OD_600_ recorded every 15 minutes for 24 hours. OD_600_ for cobamide dose–response assays was measured with the BioTek Synergy 2 microplate reader following 22-hour growth at 37°C with shaking in either the plate reader or a heated benchtop microplate shaker (1,200 rpm, Southwest Science). Preparation of cultures containing adenosylcobamides was done under red light, and the plates were incubated in the dark. EC_50_ values were calculated using GraphPad Prism (dose-response – stimulation; [agonist] vs response – variable slope (four parameters)). Growth rates and lag times were extracted from growth assay data with AMiGA (75), using 0.01 as the minimum OD_600_ value following pathlength correction to 1 cm.

For competition experiments involving evolved populations or strains, cells were precultured in M9 glycerol medium supplemented with Met. Cells were pelleted, washed twice with 0.85% saline, and resuspended in M9 glycerol medium, with the exception of the experiments shown in Fig. 2C and 3D, in which the cells were pelleted and resuspended in M9 glycerol medium without washing. OD_600_ was measured, and the population or an equal ratio of two strains was inoculated at a starting OD_600_ of 0.01 in 200 µL M9 glycerol medium containing the indicated supplement. A dilution of the culture was plated on LB agar to establish the percentage of small colonies at time point 0. The plate was sealed and incubated at 37°C in a benchtop microplate shaker at 1,200 rpm. 2 µL of each culture was transferred into 198 µL fresh medium every 24 hours. On the indicated days, dilutions from the cultures were plated on LB agar to determine the percentage of small colonies in the population.

Competition experiments involving *btuB*-overexpression strains were tracked by fluorescence (71). Strains were pre-cultured in M9 glycerol medium supplemented with Met. Cells were pelleted, washed twice with 0.85% saline, and resuspended in M9 glycerol medium. OD_600_ was measured, and each sample was adjusted to an OD_600_ of 0.25. Co-cultures were prepared by mixing an equal volume of each strain. 100 µL of each co-culture was transferred to a 96-well glass bottom plate (P96-1.5P, Cellvis), and cyan and yellow fluorescence were measured on a multiwell plate reader (Tecan Spark) as described (71). Separately, 8 µL of each mono- and co-culture were added to 192 µL of M9 glycerol medium (starting OD_600_ of 0.01) containing the specified amendment in 96-well plates. Plates were sealed and incubated at 37°C in a benchtop microplate shaker (1,200 rpm). 2 µL of each culture was transferred into 198 µL fresh medium every 24 hours. At the specified time points, aliquots were diluted in M9 medium, and CFP and YFP values were measured. Standard curves for conversion of fluorescence to OD_600_ equivalents were generated from saturated cultures grown in tubes (for t = 0 readings only) or monoculture controls grown in 96-well plates (from t = 1 day on). Competition experiments with pACYCDuet-1-pLac plasmids expressing *btuR* and/or *yciK* were performed similarly, except that strains were pre-cultured in M9 glucose (0.2%) medium with Met.

### Whole-genome sequencing and analysis

Evolved populations were grown in M9 medium supplemented with 0.35 nM pCbl, while evolved isolates and the ∆*metE* ancestor were cultured in M9 medium supplemented with Met. Genomic DNA was isolated with a DNeasy Blood and Tissue Kit (Qiagen) and submitted to Novogene (Sacramento, CA, USA) for library preparation and whole-genome sequencing using an Illumina NovaSeq 6000.

Identification of mutations was performed by Novogene by comparison to the *E. coli* MG1655 reference genome (accession PRJNA57779). SNPs and InDels were detected using SAMtools with the parameter “mpileup -m 2 F 0.002 -d 1000” and annotated using ANNOVAR (76, 77). The results were filtered such that the number of support reads for each SNP/InDel was greater than 4 and the mapping quality of each SNP/InDel was higher than 20. SVs were detected by BreakDancer and annotated by ANNOVAR (78). SVs were filtered by removing those with fewer than two supporting PE reads. A comparison to the ∆*metE* ancestor was made to eliminate mutations in the genome present prior to the laboratory evolution.

### Measurement of *btuB* and *btuR* expression in evolved isolates by RT-qPCR

The *E. coli* Δ*metE* ancestor and evolved isolates S2, S3, S4, R1, and R3 were streaked onto LB agar and grown at 37°C. Three individual colonies of each strain were inoculated into M9 glycerol medium supplemented with 1 nM pCbl. After 22 hours of growth at 37°C, the cultures were passaged into fresh medium at an OD_600_ of 0.01 and grown for 20 hours. Cultures were then diluted to an OD_600_ of 0.03 in fresh medium, and samples were collected during exponential phase (OD_600_ ~0.4). 875 µL of each culture was mixed with 1.75 mL RNAprotect Bacteria Reagent, and RNA was extracted with the RNeasy Mini Kit (Qiagen). On-column DNase digestion was performed to remove genomic DNA. RNA was quantified using a Qubit fluorometer (Invitrogen). 100 ng of RNA was used to synthesize cDNA in 20-µL reactions with iScript RT Supermix (Biorad). 5 µL of 50-fold diluted cDNA was used in 20-µL reactions with SsoAdvanced Universal SYBR Green Supermix (Biorad), with reactions performed in triplicate with each primer pair (5 µM). qPCR was performed on a CFX96 Touch Real-Time PCR Detection System (Biorad) with the following conditions: one cycle of denaturation at 95°C for 3 minutes, and 40 cycles of denaturation at 95°C for 10 seconds and extension at 59°C for 30 seconds. C_q_ values were normalized against previously reported reference genes *mdh* and *rpoA* (79), and relative gene expression was calculated using the ΔΔC_q_ method. Primers used for qPCR are listed in Table S2.

### HPLC analysis of cellular methylcobamides and adenosylcobamides

*E. coli* strains MG1655 pACYCDuet-1-pLac (*btuR* WT), MG1655 pACYCDuet-1-pLac-*btuR* (*btuR* OE), and ∆*btuR* pACYCDuet-1-pLac (∆*btuR*) were pre-cultured overnight in 35 mL M9 glucose medium supplemented with Cm. Cells were pelleted and washed once with saline and inoculated into 250 mL M9 glycerol with Cm at ~0.1 OD_600_ supplemented with either 1 nM CNpCbl or CNCbl (250 pmol). Cultures were incubated at 37°C with aeration for 6 hours before cells were pelleted and washed once with saline. Corrinoids were extracted in the absence of KCN as described (26). Extractions were analyzed on an Agilent Zorbax SB-Aq column (5 µm, 4.6 × 150 mm) with an Agilent 1200 series HPLC equipped with a diode array detector using Method 2 (10). MepCbl/MeCbl and AdopCbl/AdoCbl were quantified by comparison of peak areas (525 nm) to injections of known quantities of MeCbl or AdoCbl, respectively. The extraction efficiency of pure MeCbl and AdoCbl with Sep-Pak C18 cartridges (Waters) is ≥85%.

## SUPPLEMENTAL MATERIAL

Supplemental figures jb.00284-24-s0001.docx Fig. S1 to S7.

Table S1 jb.00284-24-s0002.xlsx Mutations in evolved isolates and populations.

Table S2 jb.00284-24-s0003.docx qPCR primers.

ASM does not own the copyrights to Supplemental Material that may be linked to, or accessed through, an article. The authors have granted ASM a non-exclusive, world-wide license to publish the Supplemental Material files. Please contact the corresponding author directly for reuse.
