# Supplementary material for: Laboratory evolution of E. coli with a natural vitamin B12 analog reveals roles for cobamide uptake and adenosylation in methionine synthase-dependent growth
Source: J Bacteriol. 2025 Jan 28;207(2):e00284-24. doi: 10.1128/jb.00284-24 (PMC11841063; doi:10.1128/jb.00284-24)
Supplement: Supplemental figures — Fig. S1 to S7. [file jb.00284-24-s0001.docx]

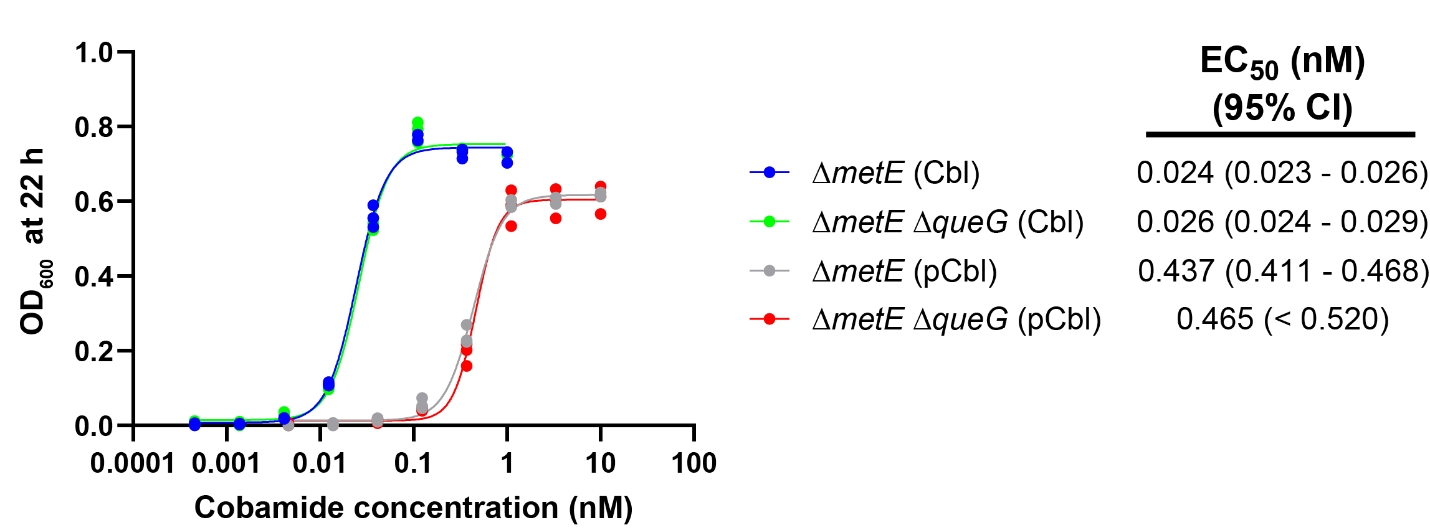


**Figure S1. Deletion of *queG* does not affect ∆*metE*** **phenotype.** Dose-response curves of *E. coli* ∆*metE* and ∆*metE* ∆*queG* strains grown in the absence of methionine with various concentrations of Cbl or pCbl. OD_600_ was recorded after 22 hours. EC_50_ values and 95% confidence intervals of three biological replicates for each cobamide were calculated in GraphPad Prism (four-parameter non-linear fit); less than symbol indicates that the lower bound of the confidence interval could not be determined.


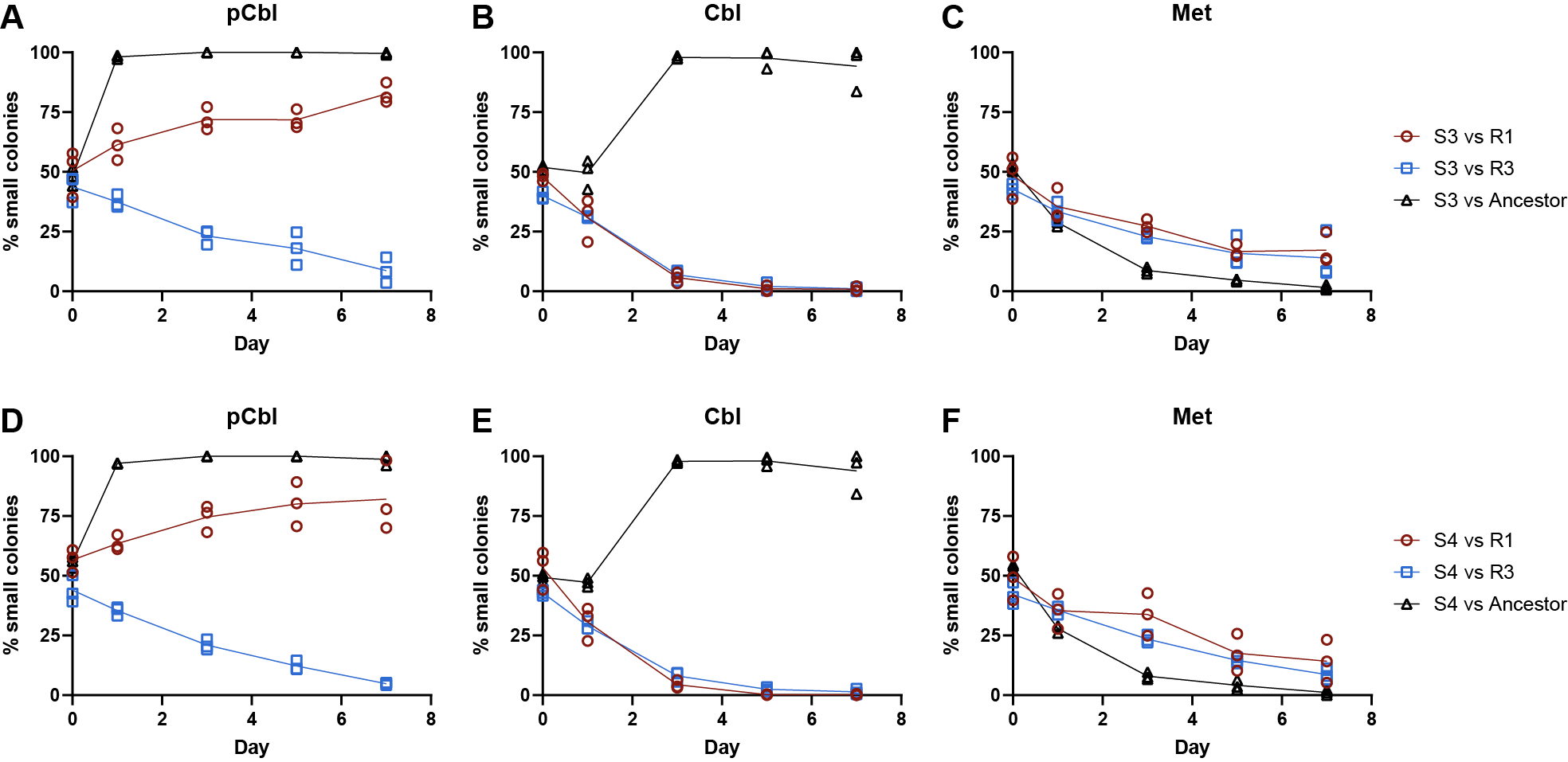


**Figure S2.** **Growth competition of small and regular sized isolates from Culture 8.** Isolates S3 (A-C) and S4 (D-F) were competed in co-culture against isolates R1 and R3 and the ancestor strain with daily passaging in medium containing either 0.35 nM pCbl, 0.35 nM Cbl, or 0.1 mg/ml Met. Cultures were diluted and plated on the indicated days to quantify the fraction of small colonies (S3 or S4). Lines connect the means of three biological replicates.


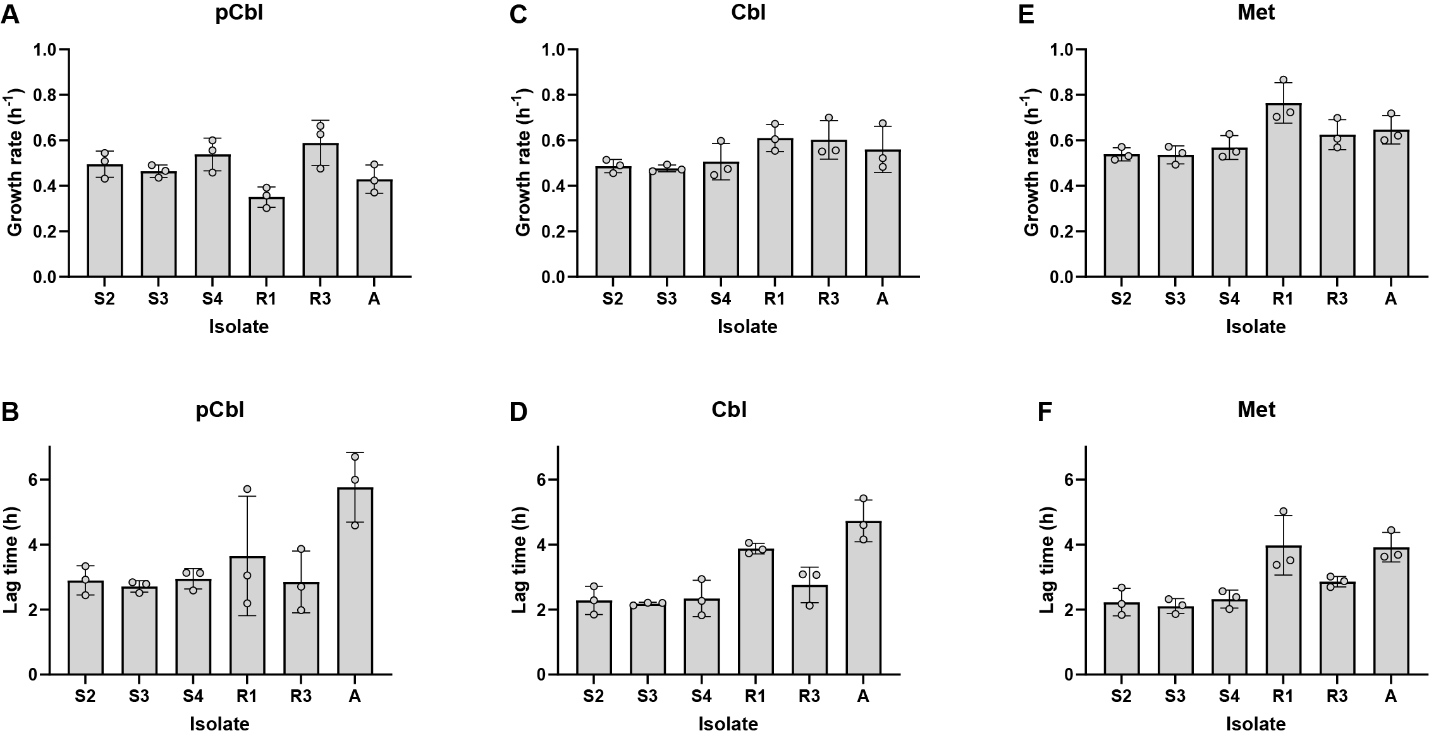


**Figure S3.** **Growth rates and lag times of evolved isolates and ancestor strain grown in pure culture.** Growth rates and lag times were calculated from the growth curves shown in Fig. 3E-G for the evolved isolates S2, S3, S4, R1, and R3, and the ancestor strain, labeled A. Bars represent the means of the growth parameters calculated individually for each of three replicates (shown as open circles), with error bars representing standard deviation.


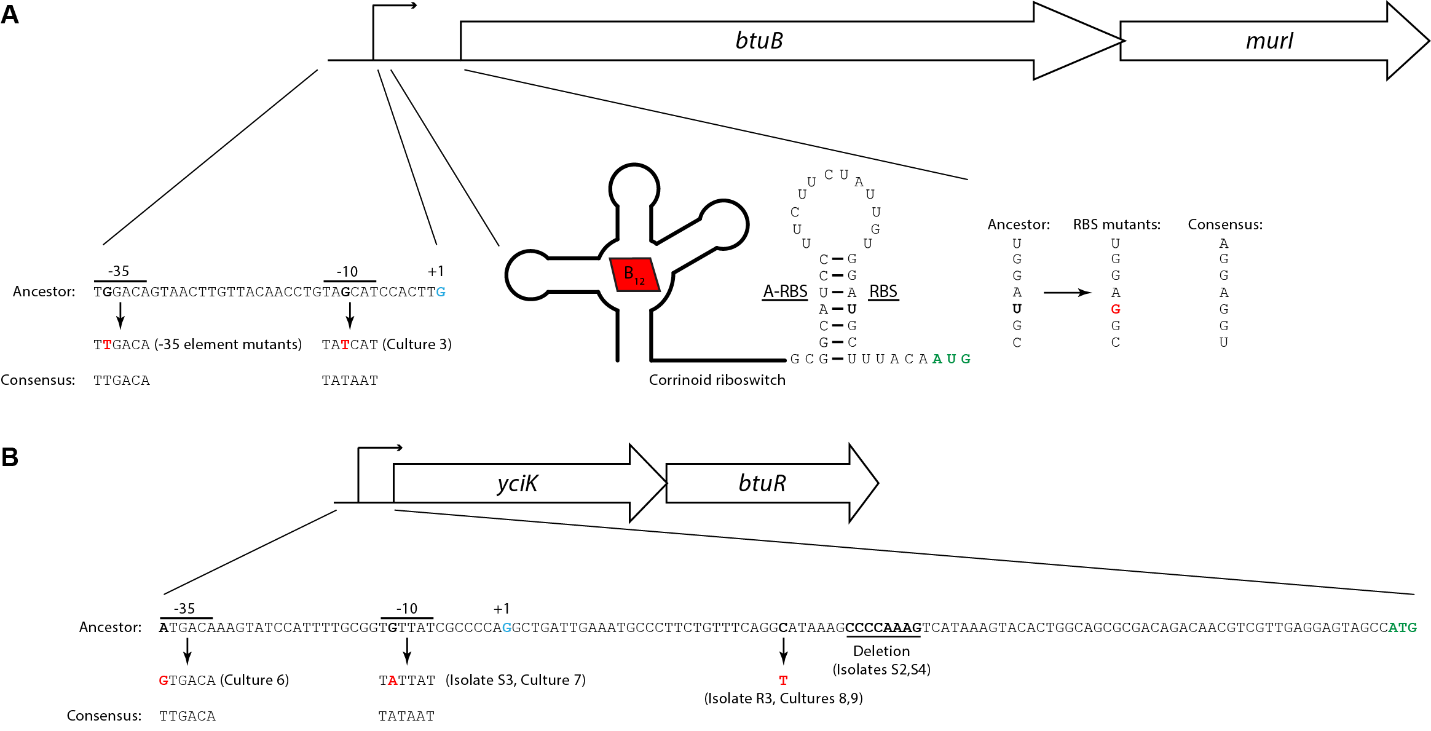


**Figure S4. Mutations in the *btuB-murI* and *yciK-btuR* operons in evolved isolates and populations.** Nucleotides in the native sequence that were mutated are bolded, with their corresponding changes shown in red. Transcriptional start sites and start codons are shown in blue and green, respectively. The consensus sequences for the σ^70^ promoter -35 and -10 elements, and RBS are shown for comparison. The promoter for the *yciK-btuR* operon has not been experimentally characterized and was predicted by PromoterHunter. A) Changes in the promoter and RBS/corrinoid riboswitch of the *btuB-murI* operon likely cause increased expression. The mutations found in the -35 element, -10 element, and RBS result in sequences closer to their respective consensus sequences. Additionally, the mutation in the RBS may disrupt its transcription- and translation-inhibiting interaction with the anti-RBS (A-RBS) within the corrinoid riboswitch. B) Changes to the promoter and 5ʹ UTR of the *yciK-btuR* operon. *yciK-btuR* is on the minus strand of the chromosome and the reverse complement of the promoter and 5ʹ UTR is shown. The mutation found in the -10 element likely increases transcription, as the sequence is closer to the consensus sequence. It is unclear how the other mutations affect expression of the operon.


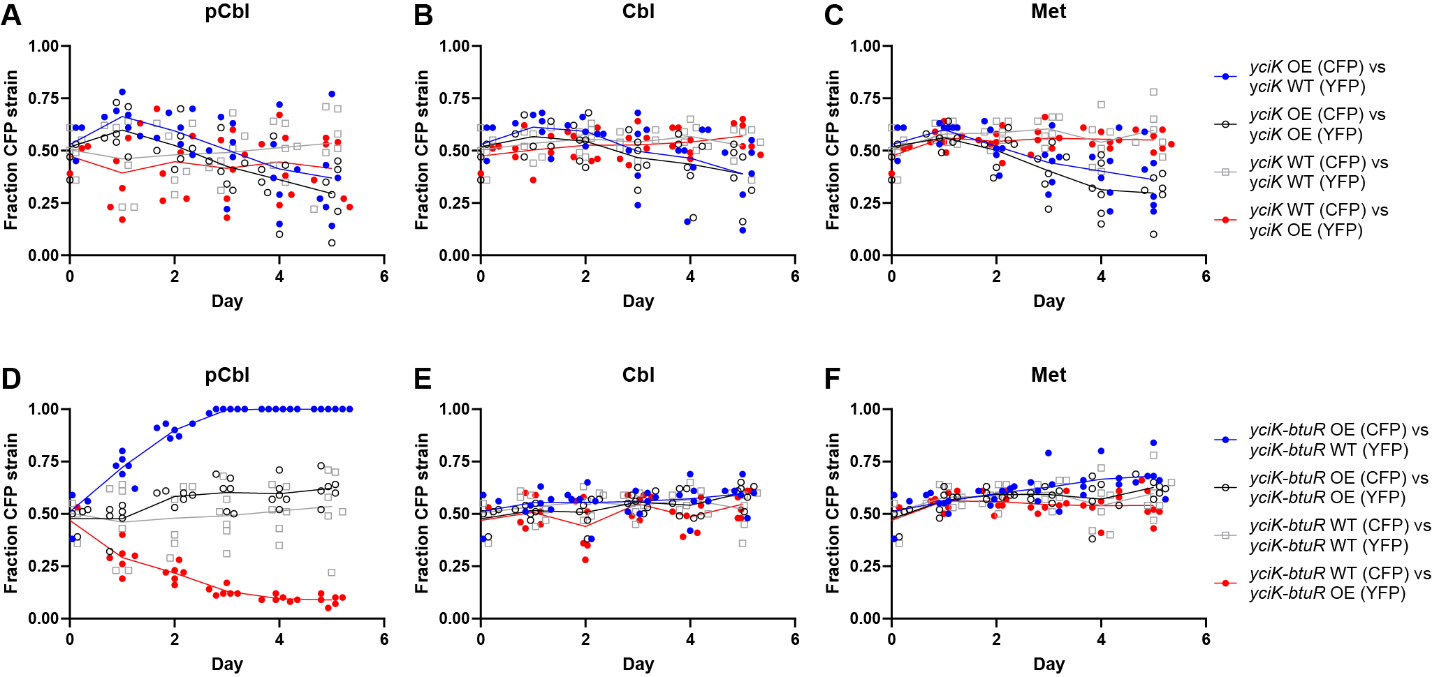


**Figure S5. Overexpression of** ***yciK* does not confer a growth advantage with pCbl.** CFP- and YFP-expressing ∆*metE* strains overexpressing *yciK* (OE) or producing native levels of *yciK* (WT) (A-C), and ∆*metE* strains overexpressing *yciK-btuR* (OE) or producing native levels of *yciK-btuR* (WT) (D-F), were competed in co-culture with daily passaging for five days in medium containing either 1 nM pCbl, 1 nM Cbl, or 0.1 mg/ml Met. The fraction of the CFP-expressing strain in each co-culture is plotted. Control co-cultures containing CFP- and YFP-expressing strains in the same genetic background (black and gray) were included to rule out a growth disadvantage caused by either fluorescent protein. Lines connect the means of six biological replicates.


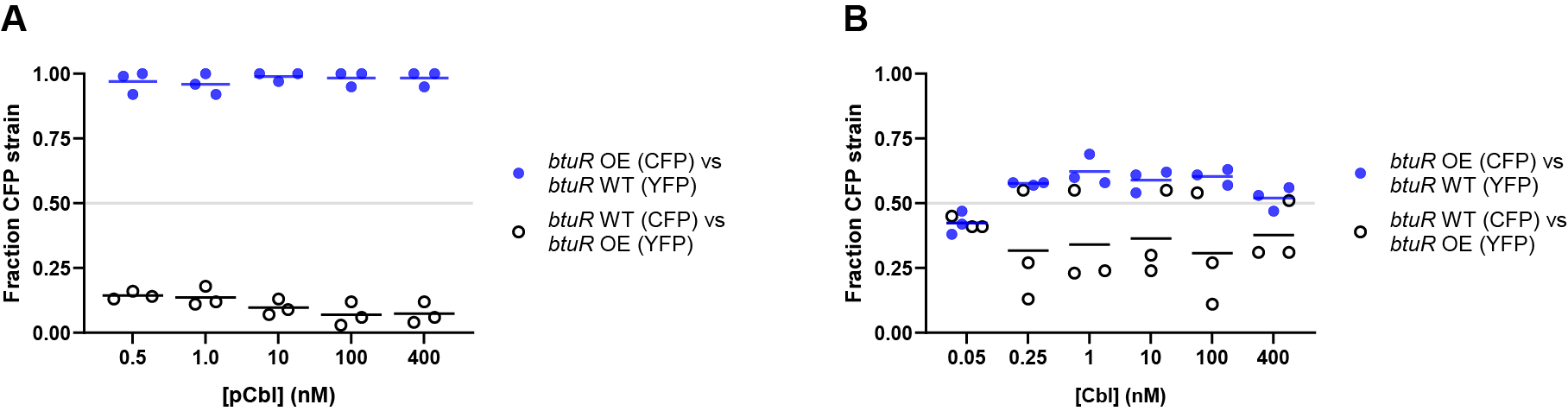


**Figure S6.** **Overexpression of *btuB* does not affect *btuR* phenotype.** A-B) The CFP- and YFP-expressing strains that overexpress *btuR* (OE) or produce native levels of *btuR* (WT) in the *btuB*-overexpression background were competed in co-culture at different concentrations of pCbl (A) or Cbl (B). Fluorescence was measured on day 3 following daily passaging. Lines represent the means of three biological replicates.


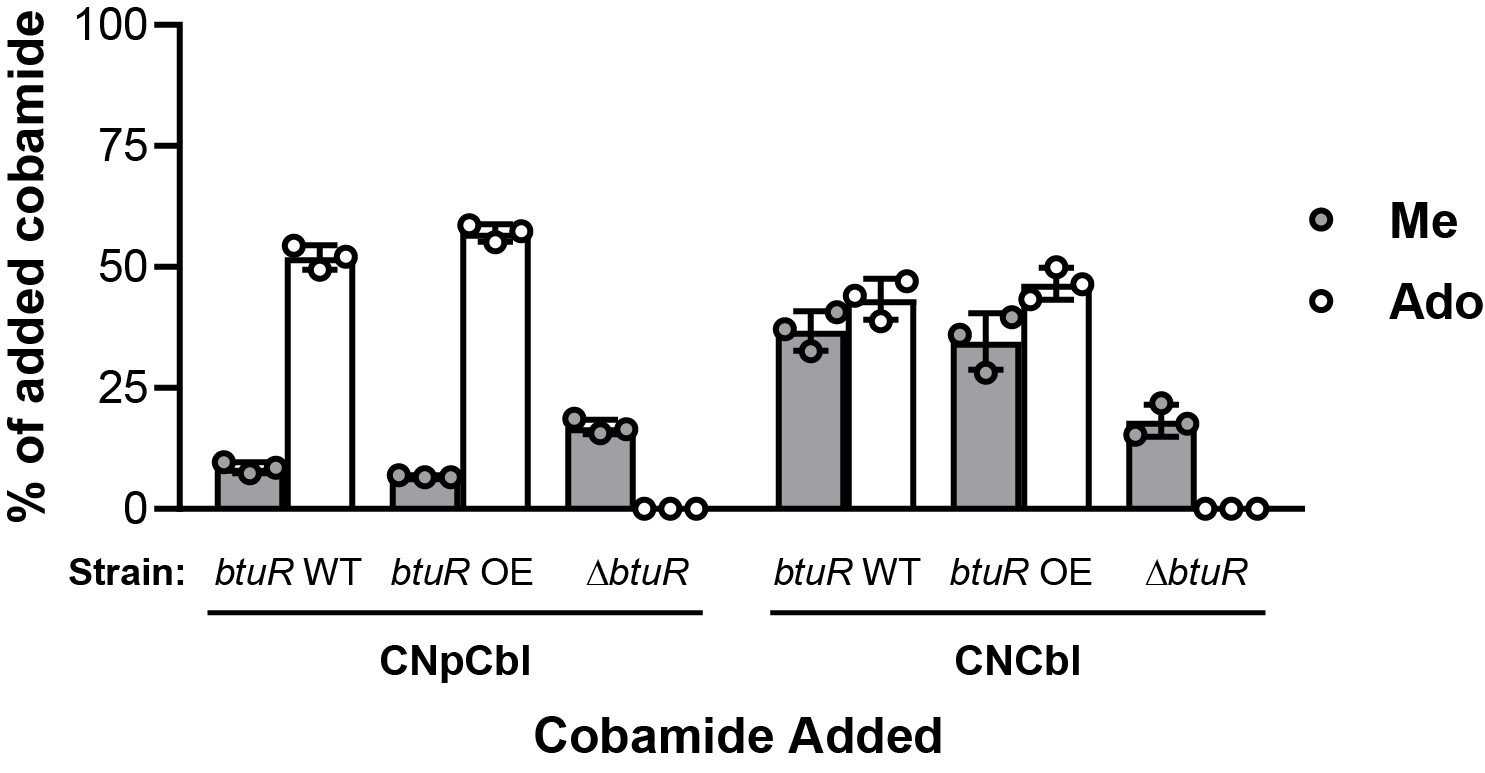


**Figure S7. Overexpression of *btuR* does not alter levels of methyl- or adenosylcobamides.** Methyl (Me, gray) and adenosyl (Ado, white) cobamides were extracted from MG1655 pACYCDuet-1-pLac (*btuR* WT), MG1655 pACYCDuet-1-pLac-*btuR* (*btuR* OE), and MG1655 *∆btuR* pACYCDuet-1-pLac (*∆btuR*) cells incubated with either CNpCbl or CNCbl, and analyzed by HPLC. Cobamide levels were quantified by comparing to standards of MeCbl and AdoCbl and normalized to the amount of cobamide added to the culture (250 pmol). Data represent the average and standard deviation of three biological replicates. The extraction efficiency of pure MeCbl and AdoCbl with the C18 cartridges used in the extraction is ≥85%, but has not been tested with MepCbl or AdopCbl. Therefore, only levels of pCbl with the same upper ligand should be compared to each other in strains grown with CNpCbl.
