# Supplementary material for: Laboratory evolution of E. coli with a natural vitamin B12 analog reveals roles for cobamide uptake and adenosylation in methionine synthase-dependent growth
Source: J Bacteriol. 2025 Jan 28;207(2):e00284-24. doi: 10.1128/jb.00284-24 (PMC11841063; doi:10.1128/jb.00284-24)
Supplement: Table S2 — qPCR primers. [file jb.00284-24-s0003.docx]

**Table S2. qPCR primers**

| **Primer** | **Sequence** | **Primer efficiency** |
| --- | --- | --- |
| mdh-F | CGGTTATTGGCGGTCACTCT | 95.8% (r^2^ = 0.997) |
| mdh-R | CGTTCTGGATGCGTTTGGTC |  |
| rpoA-F | GGAAGAAGATGAGCGCCCAA | 99.2% (r^2^ = 0.999) |
| rpoA-R | CGCGCTGCTTCAACATTGTA |  |
| btuB-F | TGGTCGTTATGATTCGTCGG | 98.0% (r^2^ = 0.999) |
| btuB-R | GTCGTAGTCTGTTTCTGCCA |  |
| btuR-F | TCAGCAGCGAGTGAAAGAAA | 99.8% (r^2^ = 0.993) |
| btuR-R | TTTTGCCTTTTCCATTGCCG |  |
